# Structural insights into nanoRNA degradation by human Rexo2

**DOI:** 10.1261/rna.070557.119

**Published:** 2019-06

**Authors:** Lee-Ya Chu, Sashank Agrawal, Yi-Ping Chen, Wei-Zen Yang, Hanna S. Yuan

**Affiliations:** 1 Institute of Molecular Biology, Academia Sinica, Taipei, Taiwan 11529, Republic of China; 2 Chemical Biology and Molecular Biophysics Program, Taiwan International Graduate Program, Academia Sinica, Taipei, Taiwan 11529, Republic of China; 3 Institute of Bioinformatics and Structural Biology, National Tsing Hua University, Hsin Chu, Taiwan 30013, Republic of China; 4 Molecular and Cell Biology Program, Taiwan International Graduate Program, Academia Sinica, Taipei, Taiwan 11529, Republic of China; 5 Graduate Institute of Life Sciences, National Defense Medical Center, Taipei, Taiwan 11490, Republic of China

**Keywords:** crystal structure, RNA decay, ribonuclease, exonuclease, protein–RNA interactions

## Abstract

Human RNA exoribonuclease 2 (Rexo2) is an evolutionarily conserved 3′-to-5′ DEDDh-family exonuclease located primarily in mitochondria. Rexo2 degrades small RNA oligonucleotides of <5 nucleotides (nanoRNA) in a way similar to *Escherichia coli* Oligoribonuclease (ORN), suggesting that it plays a role in RNA turnover in mitochondria. However, how Rexo2 preferentially binds and degrades nanoRNA remains elusive. Here, we show that Rexo2 binds small RNA and DNA oligonucleotides with the highest affinity, and it is most robust in degrading small nanoRNA into mononucleotides in the presence of magnesium ions. We further determined three crystal structures of Rexo2 in complex with single-stranded RNA or DNA at resolutions of 1.8–2.2 Å. Rexo2 forms a homodimer and interacts mainly with the last two 3′-end nucleobases of substrates by hydrophobic and π−π stacking interactions via Leu53, Trp96, and Tyr164, signifying its preference in binding and degrading short oligonucleotides without sequence specificity. Crystal structure of Rexo2 is highly similar to that of the RNA-degrading enzyme ORN, revealing a two-magnesium-ion-dependent hydrolysis mechanism. This study thus provides the molecular basis for human Rexo2, showing how it binds and degrades nanoRNA into nucleoside monophosphates and plays a crucial role in RNA salvage pathways in mammalian mitochondria.

## INTRODUCTION

RNA decay and surveillance play key roles in gene expression regulation and RNA quality control (Moraes 2010). In human mitochondria, irregular RNA processing and decay are frequently associated with adverse pathologic conditions, including inflammation and aging (Rorbach and Minczuk 2012). A number of human ribonucleases and helicases participate in mitochondrial RNA (mtRNA) decay, including polynucleotide phosphorylase (PNPase), Suv3 helicase and RNA exoribonuclease 2 (Rexo2, also named small fragment nuclease, SFN). PNPase forms a complex with the helicase Suv3 to cooperatively degrade mtRNA with secondary structures (Minczuk et al. 2002; Wang et al. 2009), producing final cleavage products of ∼4 nucleotides (nt) (Lin et al. 2012; Golzarroshan et al. 2018). In contrast, Rexo2 likely acts as a scavenger, degrading small single-stranded RNA of <5 nt (referring to as “nanoRNAs”) and generating monoribonucleotides for RNA salvage in mitochondria (Goldman et al. 2011; Bruni et al. 2013).

Rexo2 is a 3′-to-5′ exoribonuclease in the DEDDh superfamily—also named the DnaQ-like, RNase T or RNase D superfamily—which comprises thousands of members involved in various aspects of RNA and DNA processing in all kingdoms of life (Yang 2011). Rexo2 contains four strictly conserved DEDD residues (D47, E49, D147, D199) for binding two metal ions, as well as a general base (H194) in the active site for hydrolysis of the phosphodiester bonds in nucleic acid substrates. Human Rexo2 shares high sequence identities (48% and 32%, respectively) with *Escherichia coli* Oligoribonuclease (ORN) and *Saccharomyces cerevisiae* YNT20 (Nguyen et al. 2000). Bacterial ORN preferentially binds and degrades nanoRNAs and deletion of ORN in *E. coli* is lethal, signifying its essential role in cellular metabolic pathways (Zhang et al. 1998; Ghosh and Deutscher 1999). Knockdown of ORN in *E. coli* or *Pseudomonas aeruginosa* leads to accumulation of nanoRNAs and a dramatic shift in transcription start sites for a significant proportion of promoters, suggesting that these nanoRNAs may serve as primers for transcriptional initiation and affect gene expression (Goldman et al. 2011). *Pseudomonas aeruginosa* ORN also degrades 5′-phosphoguanylyl-(3′,5′)-guanosine (pGpG) into GMP to complete the final step in the degradation of cyclic-di-GMP, an important bacterial secondary messenger molecule in controlling biofilm formation and bacterial pathogenesis (Orr et al. 2015). Yeast YNT20 is required for processing U4, U5L, and U5S snRNAs and for maturation of RNA components of both RNase P and 5.8S rRNA in the nucleus (Hanekamp and Thorsness 1999; van Hoof et al. 2000). Depletion of YNT20 leads to a reduced rate of YME1-mediated DNA escape from mitochondria—a process mediated by the inner mitochondrial membrane-associated protease YME1 (Hanekamp and Thorsness 1999)—indicating that yeast YNT20 has diverse roles in both RNA and DNA metabolism in nuclei and mitochondria.

Human Rexo2 not only shares high sequence identity but also similar enzymatic activity to bacterial ORN, as Rexo2 also preferentially degrades nanoRNAs of <5 nt (Nguyen et al. 2000). Rexo2 also degrades small DNA substrates in the presence of Mn^2+^, indicating that Rexo2 may be involved in cellular deoxynucleotide recycling (Nguyen et al. 2000; Bruni et al. 2013). Rexo2 is primarily expressed with a mitochondrial localization sequence (MLS) and is localized in the mitochondrial intermembrane space and matrix, though a small amount of Rexo2 is expressed without an MLS and is localized in cytoplasm (Bruni et al. 2013). Depletion of Rexo2 in HeLa cells affects mitochondrial morphology and cell growth, leading to a substantial decrease in both mitochondrial DNA (mtDNA) and mtRNA. As mitochondrial protein synthesis is also decreased in Rexo2-depleted cells, depletion of Rexo2 likely further affects protein translation and mtDNA replication due to impairment of nanoRNA degradation (Bruni et al. 2013). Together, these results support a role for Rexo2 in degrading nanoRNAs to provide nucleoside monophosphates for RNA synthesis in mitochondria. A number of crystal structures of bacterial ORN were reported, including those from *Xanthomonas campestris* (Chin et al. 2006) (PDB code: 2GBZ), *Coxiella burnetii* (Franklin et al. 2015) (PDB codes: 3TR8), and *Colwellia psychrerythraea* (Lee et al. 2019) (PDB code: 6A4A, 6A4D, 6A4E), or deposited in the protein data bank, including those from *E. coli* (PDB codes: 1YTA, 2IGI), *Acinetobacter baumannii* (PDB code: 5CY4), and *Haemophilus influenzae* (PDB code 1J9A). However, the molecular basis of how Rexo2 preferentially binds and degrades nanoRNAs remains elusive.

Here, we show that Rexo2 preferentially degrades nanoRNAs of 2–4 nt in the presence of Mg^2+^ because it binds nanoRNAs with the highest affinities. We determined three crystal structures of Rexo2 (wild-type or D199A mutant) in complex with single-stranded RNA or DNA in the presence of Mg^2+^ at resolutions of 1.8–2.2 Å. Rexo2 interacts primarily with the last 2 nt bases at the 3′ end of nucleic acid substrates via hydrophobic and π−π stacking interactions. Thus, our results provide the molecular basis for the preference of Rexo2 for degrading small oligonucleotides by a two-Mg^2+^-dependent hydrolysis mechanism in mitochondria.

## RESULTS

### Rexo2 preferentially binds and degrades small RNAs into mononucleotides

**FIGURE 1.**
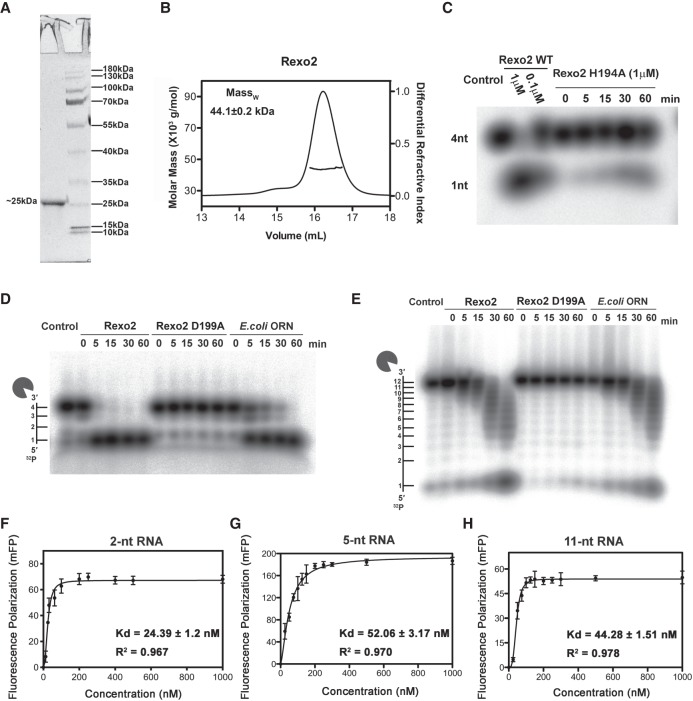
Rexo2 is a robust ribonuclease that degrades small RNA oligonucleotides into mononucleotides. (*A*) Recombinant Rexo2 purified to high homogeneity, as revealed by SDS-PAGE. (*B*) Rexo2 forms a homodimer with a MW of ∼44 kDa, as estimated by size exclusion chromatography-coupled multiangle light scattering (SEC-MALS). (*C*) The Rexo2–H194A mutant exhibits only residual RNase activity in degrading a 4-nt RNA relative to that of wild-type Rexo2. (*D*,*E*) Wild-type Rexo2 (1 µM) and *E. coli* ORN (50 nM) degrades 4-nt RNA (5′-^32^P-A_4_-3′) or 12-nt RNA (5′-^32^P-A_12_-3′) into mononucleotides in the presence of 5 mM MgCl_2_, whereas the Rexo2–D199A mutant (1 µM) exhibits only residual RNase activity. (*F*–*H*) RNA binding affinities between Rexo2–H194A and 2-nt RNA (5′-Cy3-A_2_-3′), 5-nt RNA (5′-Cy3-A_5_-3′), and 11-nt RNA (5′-Cy3-AGCGCAGUACC-3′) substrates were measured by fluorescence polarization (in mFP units) and plotted against protein concentrations. The RNA-binding affinities of Rexo2 were calculated by fitting the binding curve to a one-site-binding Hill slope, giving estimated Hill coefficients of 2.3, 1.4, and 3.5 for 2-nt, 5-nt, and 11-nt RNA, respectively. See also Supplemental Figure S1.

To understand how Rexo2 binds and degrades RNA, we first expressed wild-type His-tagged Rexo2 without the amino-terminal MLS and carboxy-terminal tail in *E. coli*. For RNA-binding and cocrystallization experiments, we expressed an additional two inactive Rexo2 mutants; Rexo2–D199A in which the metal ion-binding residue is replaced and Rexo2–H194A in which the general base residue is replaced. We subsequently removed the His-tag of recombinant Rexo2 proteins using Thrombin. Final purified Rexo2 proteins, containing only residues 33–223, were stable dimers with a molecular weight (MW) of ∼44 kDa (calculated MW for a single protomer: 22.436 kDa, Fig. 1A,B).

To assess RNase activity, we incubated Rexo2 and Rexo2–D199A (1 μM) with either 5′-end-^32^P-labeled 4-nt RNA (5′-^32^P-A_4_-3′) or 12-nt RNA (5′-^32^P-A_12_-3′) in the presence of 5 mM MgCl_2_. For comparison, we expressed and purified *E. coli* ORN according to a previously described procedure (Fiedler et al. 2004), and incubated it with the two RNA substrates. Comparing to ORN (50 nM), a higher concentration of Rexo2 (1 μM) cleaved RNA with similar efficiencies, suggesting that Rexo2 has a weaker ribonuclease activity in degrading RNA (Fig. 1D). Rexo2 efficiently degraded the 4-nt RNA into mononucleotides, with little signal of intermediate bands representing 3-nt and 2-nt fragments as seen in the time-course experiments (Fig. 1D). Rexo2 also degraded longer 12-nt RNAs but with lower activity, evidenced by the appearance of mid-sized bands representing longer RNAs of 4–11 nt (Fig. 1E). Rexo2 shared a similar preference to ORN for degrading nanoRNA, whereas Rexo2–D199A and Rexo2–H194A had no or only residual activity in terms of degrading RNAs (Fig. 1C–E). These results confirm earlier reports showing that Rexo2 preferentially degrades nanoRNAs of <5 nt (Nguyen et al. 2000; Bruni et al. 2013).

Human Rexo2 has been shown to degrade small DNA oligonucleotides in the presence of Mn^2+^ (Bruni et al. 2013). To test the DNase activity of Rexo2 in the presence of Mg^2+^, we incubated Rexo2 and Rexo2–D199A with 5′-end-^32^P-labeled 4-nt DNA (5′-^32^P-T_4_-3′) and 12-nt DNA (5′-^32^P-T_12_-3′) in the presence of 5 mM MgCl_2_. We used a higher concentration of Rexo2 (2 μM) in this instance (relative to the RNase activity assay), as its DNA degradation activity is lower than that for RNA. Consistent with the earlier finding (Nguyen et al. 2000), we found that Reox2 has an approximately fourfold higher catalysis rate for degrading RNA over DNA. We observed a similar trend to RNA degradation in that Rexo2 degraded 4-nt DNA more efficiently than 12-nt DNA (Supplemental Fig. S1). We also noted that Rexo2 had almost no DNase activity in degrading single-stranded DNA of poly(A) or poly(G) in the presence of Mg^2+^ (data not shown). Compared to Rexo2, ORN (2 μM) exhibited lower DNase activity for degrading either 4-nt or 12-nt DNA (Supplemental Fig. S1).

To investigate the molecular basis for the substrate preference of Rexo2, we next measured by fluorescence polarization the binding affinity of the inactive H194A Rexo2 mutant for a short fluorophore-labeled 2-nt RNA, a mid-length 5-nt RNA and a long 11-nt RNA. The inactive Rexo2–H194A mutant bound the short 2-nt 5′-Cy3-labeled RNA with the highest affinity (*K*_d_ = 24.4 nM), whereas it bound the 5-nt and 12-nt RNAs with similar lower affinities (*K*_d_ = 52.1 and 44.3 nM, respectively) (Fig. 1F–H). This result suggests that Rexo2 binds its preferred substrate, a dinucleotide, with the highest affinity and it binds longer oligonucleotides (≥5 nt) with a slightly lower affinity. Similar results were observed for DNA binding in that Rexo2 bound a short 3-nt 5′-Cy3-labeled DNA with the highest affinity (*K*_d_ = 29.9 nM), whereas it bound a mid-length 5-nt DNA and a long 12-nt DNA with lower affinities (*K*_d_ = 51.8 and 52.2 nM, respectively) (Supplemental Fig. S1). Previous studies showed that Rexo2 binds RNA and DNA with a similar *K*_m_ but degrades RNA with a higher catalysis rate over DNA (Nguyen et al. 2000). The approximately twofold higher affinity of Rexo2 toward short RNAs as compared to long RNAs may partially explain its higher cleavage efficiency for nanoRNA, but other factors, including a lower energy of transition state and a faster product releasing rate, may also contribute to the higher catalysis rate for nanoRNA substrates. Therefore, we conclude that Rexo2 preferentially binds nanoRNAs (≤4 nt) and degrades them by robust RNase activity.

### Crystal structures of Rexo2–RNA and Rexo2–DNA complexes

**FIGURE 2.**
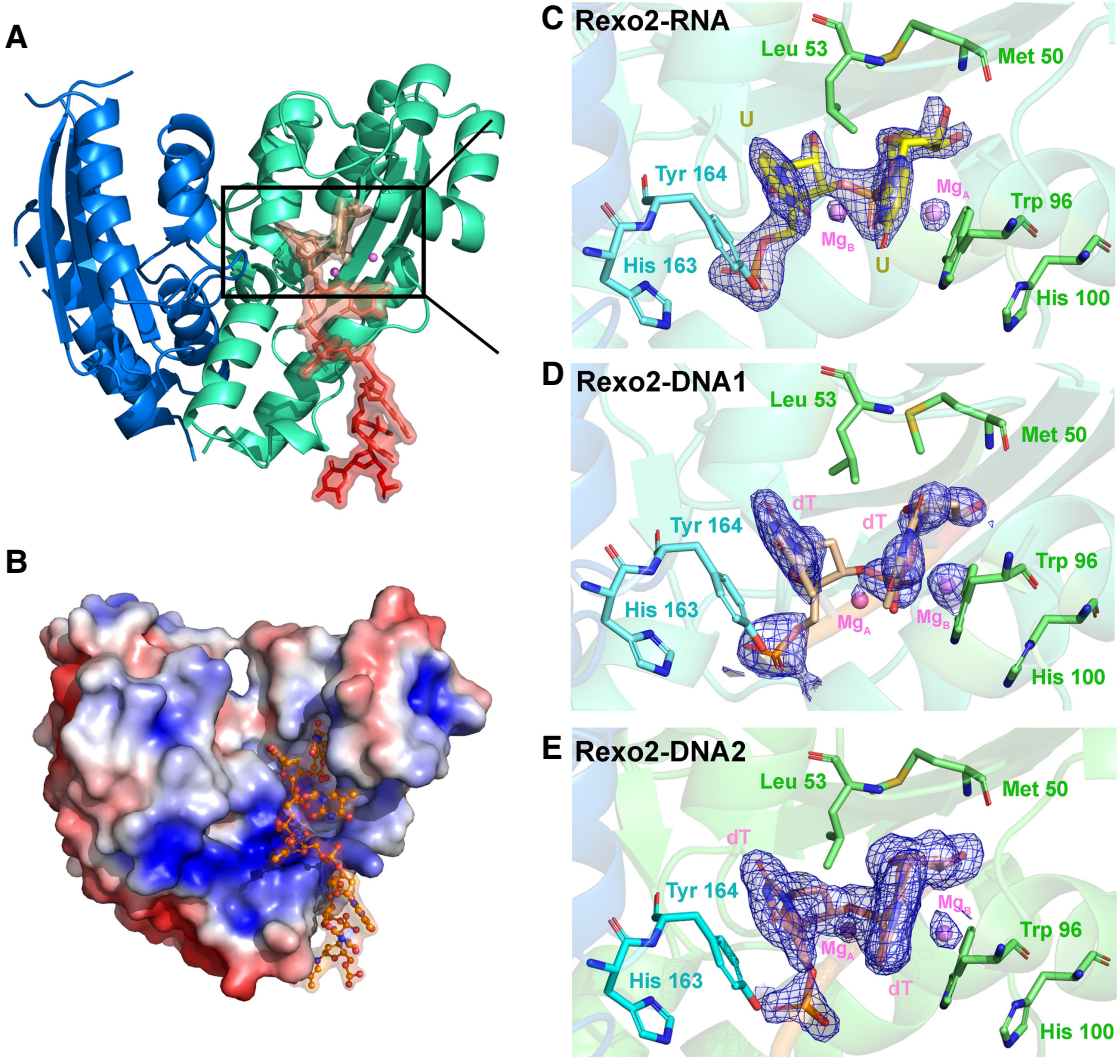
The crystal structures of the Rexo2–RNA and Rexo2–DNA complexes show how Rexo2 forms a homodimer and binds oligonucleotides. (*A*) The overall structure of the Rexo2–DNA2 complex. Rexo2 forms a homodimer, one protomer (chain B) displayed in green and the other (chain A) in blue, with one DNA strand and two Mg^2+^ ions bound in the active site of protomer B. (*B*) The electrostatic surface potential of the Rexo2–DNA2 complex reveals positive surfaces extending from the active site (red, −5.0 kBT/e; blue, +5.0 kBT/e; kB, Boltzmann constant; T, temperature in Kelvin; e, charge of an electron). (*C*–*E*) The omit electron density maps for the last two 3′-end nucleotides bound in the active site of protomer B in Rexo2–RNA complex (1.0 σ), Rexo2–DNA1 complex (0.8 σ), and Rexo2–DNA2 complex (1.0 σ). See also Supplemental Figures S2, S3.

To understand how Rexo2 binds and degrades small oligonucleotides, we next cocrystalized human Rexo2 with single-stranded RNA or DNA substrates. We screened the cocrystallization conditions for Rexo2 and the Rexo2–D199A mutant in complex with various DNA and RNA substrates in the presence or absence of Mg^2+^. We obtained three complex cocrystals, referred to as Rexo2–RNA (Rexo2–D199A bound with a 12-nt U_12_ RNA and Mg^2+^), Rexo2–DNA1 (wild-type Rexo2 bound with a 5-nt T_5_ DNA), and Rexo2-DNA2 (Rexo2–D199A bound with a 10-nt T_10_ DNA and Mg^2+^). The crystal structures of these three complexes were determined by molecular replacement using the crystal structure of *E. coli* ORN (PDBID: 2IGI) as the initial search model. Due to ill-defined electron density at the 5′ ends of RNA or DNA fragments, we only modeled partial 3′-end nucleotides in our complex structures: 2 nt (U–U) in the Rexo2–RNA complex, 2 nt (T–T) in the Rexo2–DNA1 complex, and seven (T_7_) nucleotides in the Reox2–DNA2 complex (see the omit maps of the bound substrates in Fig. 2C–E). The electron density for DNA oligonucleotides in Rexo2–DNA1 was broken (see Fig. 2D), likely because wild-type Rexo2 was used here for cocrystallization with the partially degraded DNA. All three Rexo2 complex structures contained two molecules, i.e., a dimer, in one asymmetric unit. The detailed data collection and refinement statistics are listed in Table 1.

**TABLE 1.**
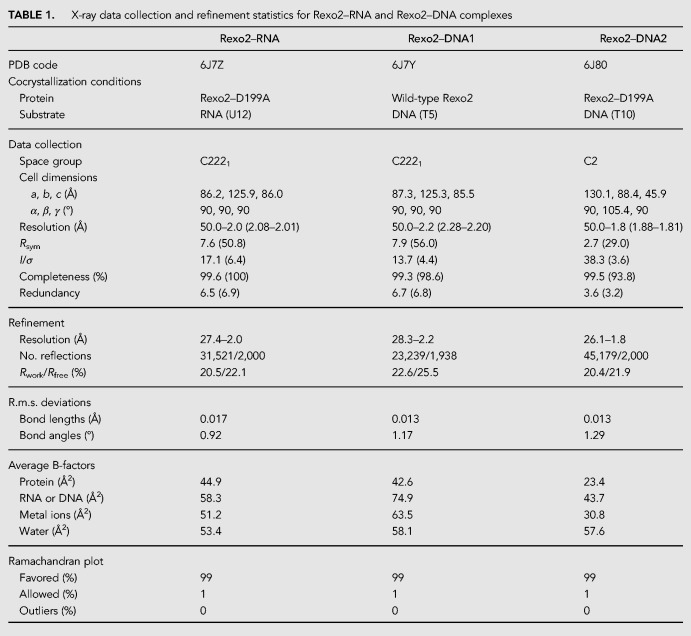
** **X-ray data collection and refinement statistics for Rexo2–RNA and Rexo2–DNA complexes

Rexo2 formed a homodimer with two protomers (chain A and B) in all three complexes, with a dimeric assembly similar to that of ORN dimer (Fig. 2A). The dimeric interfaces contain hydrophobic residues Ser170, Leu175, Trp179, Ile214, and Phe215 in α8 α9, and α10 helices, and these residues are conserved in the interfaces of ORN dimer (see Supplemental Figs. S2, S3). Protomers A and B share a similar conformation with an average RMSD of 0.52 Å (for 123 Cα atoms) in Rexo2–RNA, 0.41 Å (for 116 Cα atoms) in Rexo2–DNA1 and 0.14 Å (for 107 Cα atoms) in Rexo2–DNA2. However, several regions in protomer A were disordered, including residues 51–59, 85–114, and 190–193, in the Rexo2–RNA structure. As a result, the RNA substrate was modeled only in the active site of protomer B, but not modeled in protomer A due to the ill-defined electron density. Similarly, in Rexo2–DNA1 and Rexo2–DNA2 complex structures, DNA substrates were bound only in the active site of protomer B but not observed in protomer A.

**FIGURE 3.**
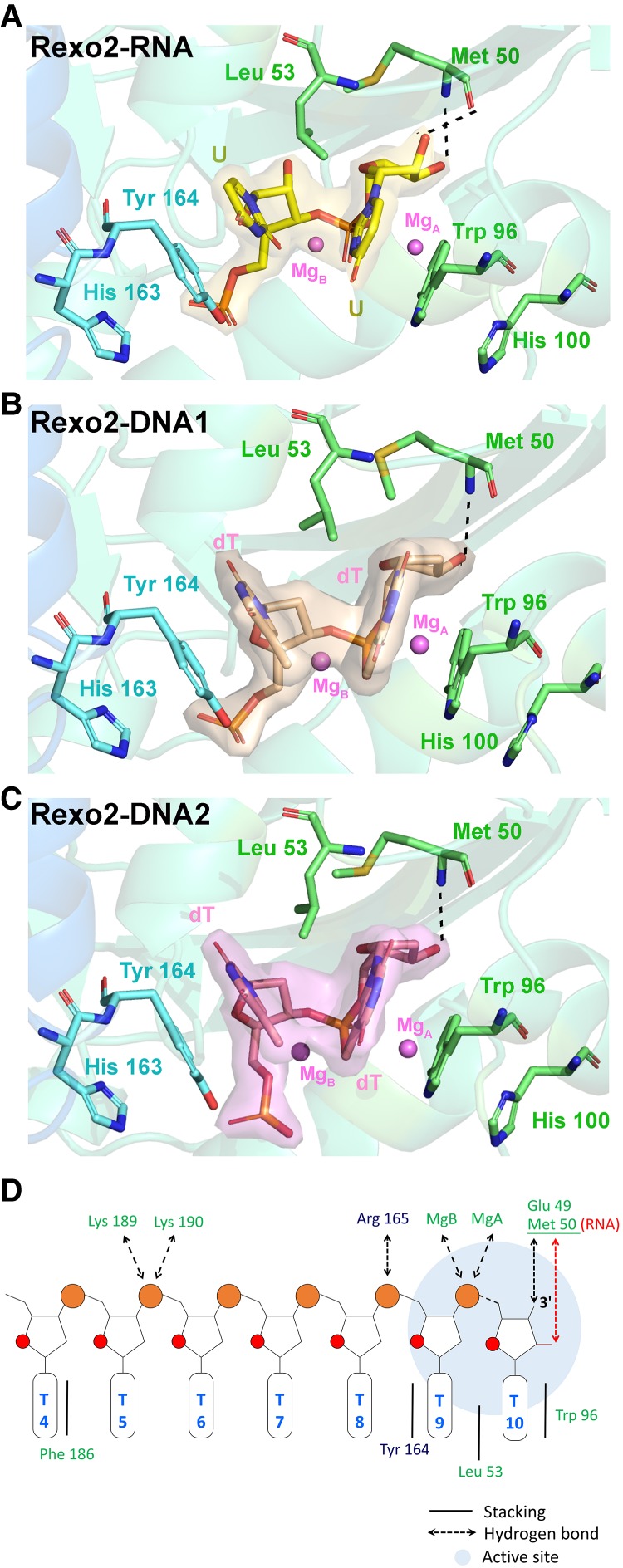
Rexo2 interacts with the last two 3′-end nucleobases of substrate by hydrophobic and π−π stacking interactions. (*A*–*C*) The last two 3′-end nucleobases are sandwiched between Tyr164, Leu53, and Trp96 in the Rexo2–RNA, Rexo2–DNA1, and Rexo2–DNA2 complexes via hydrophobic and π–π stacking interactions. (*D*) Schematic diagram for the interactions between Rexo2 and DNA in the Rexo2–DNA2 complex. The DNA-interacting residues in protomer B of Rexo2 are displayed in green, whereas the residue in protomer A (Arg165) is displayed in black. In the Rexo2–RNA complex, the hydrogen bond between 2′-OH (O2′) of the ribose group of the 3′-end nucleotide and the Met50 backbone (O atom) is displayed in red dashed line. See also Supplemental Figure S4.

The 3′ end of the RNA or DNA chain was bound in the DEDDh active site in one of the protomers (chain B) together with two Mg^2+^ ions, even though one of the metal-binding residues, D199, was mutated to Ala in two of the complexes. In the Rexo2–RNA and Rexo2–DNA1 complexes, only the last two 3′-end nucleotides were observed, i.e., U–U in Rexo2–RNA and T–T in Rexo2–DNA1, with the remainder of the substrates being disordered. In contrast, 7 nt (T4–T10) were observed in Rexo2–DNA2 complex, with additional interactions of the phosphate backbone (P5) with side chains of Lys189 and Lys190, and π–π stacking interactions between T4 nucleobase and Phe186 (Fig. 3D). Due to crystal packing, we also noted the π–π stacking interactions between T5 nucleobase and Tyr122 of the neighboring Rexo2 molecule (see Supplemental Fig. S4). The electrostatic surface of Rexo2 reveals a basic surface extending from the active site within which lie the two lysine residues (Lys189 and Lys190), suggesting that Rexo2 also binds long oligonucleotides for degradation (Fig. 2B). However, based on comparison of the three complex structures, Rexo2 appears to interact primarily with the last two 3′-end nucleotides, providing the structural basis for its preference for binding and degrading small oligonucleotides.

### Rexo2 interacts with the 3′-end dinucleotide bases by π–π stacking interactions

A close look at the interactions between Rexo2 and RNA/DNA revealed that the two nucleobases of the 3′-end di-nucleotide are sandwiched between three hydrophobic/aromatic side-chains of Leu53, Trp96, and Tyr164 (protomer B) in all three complexes (Fig. 3). The 3′-end nucleobase is sandwiched between Trp96 and Leu53, whereas the penultimate 3′-end nucleobase is sandwiched between Leu 53 and Tyr164, both of which are mediated by π−π stacking interactions. Moreover, Tyr164 further stacks with His163, whereas Trp96 further stacks with His100 (Fig. 3). These stacking interactions not only stablize the Rexo2-oligonucleotide complexes but also allow Rexo2 to bind and degrade nucliec acids without sequence specificity.

RNA-specific interactions are observed in the Rexo2–RNA complex, where the 2′-OH (O2′) of the ribose group of the 3′-end nucleotide forms a hydrogen bond with the Met50 backbone (O atom). This interaction likely facilitates product release for nucleoside monophosphates, so RNA is cleaved more efficiently than DNA. Moreover, the 3′-end OH group (O3′) forms a hydrogen bond with the Met50 backbone (NH atom) and Glu49 (Oδ) in all three complexes, providing the structural basis for the preference of Rexo2 for cleaving oligonucleoties with a 3′-OH end (Fig. 3A–C). In summary, Rexo2 binds nanoRNAs with 3′-OH ends primarily by base stacking interactions with the last two 3′-end nucleobases to degrade RNA in a sequence-independent manner.

### Two-Mg-ion catalytic mechanism of Rexo2

**FIGURE 4.**
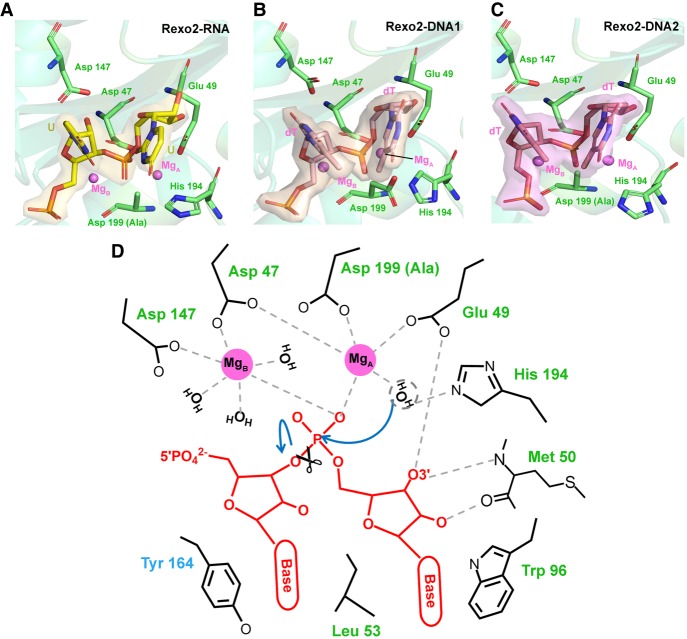
Rexo2 hydrolyzes RNA by a two-Mg^2+^-dependent mechanism. (*A*–*C*) Catalytic residues in the active site of Rexo2 in the crystal structures of Rexo2–RNA (*A*), Rexo2–DNA1 (*B*), and Rexo2–DNA2 (*C*). (*D*) Schematic diagram of the two metal-ion-dependent hydrolysis mechanism of Rexo2 responsible for degrading RNA from the 3′ end. The conserved DEDD residues, Asp47, Glu49, Asp147, and Asp199, coordinate two Mg^2+^ ions (Mg_A_ and Mg_B_), whereas the general base His194 activates a water molecule (absent from the three structures) for nucleophilic attack of the scissile phosphate. See also Supplemental Movie S1.

Our RNA and DNA degradation assays (Fig. 1; Supplemental Fig. S1) revealed that Rexo2 degrades RNA and DNA in the presence of Mg^2+^. Therefore, Rexo2 is a Mg^2+^-dependent enzyme under physiological conditions. In the Rexo2–RNA complex, we used the inactive D199A mutant for cocrystallization with RNA in the presence of Mg^2+^. D199 is one of the metal-ion binding residues in the DEDDh active site, but we still identified two Mg^2+^ ions in the active site, one bound to Glu49 (A site, Mg_A_) and another bound to Asp47 and Asp147 (B site, Mg_B_) (Fig. 4A). In the Rexo2–DNA1 complex, we used wild-type Rexo2 for cocrystallization with DNA in the absence of Mg^2+^. However, again, we still observed two metal ions (with a low occupancy of 0.81 for Mg_A_ and 0.73 for Mg_B_) bound in the active site, likely representing endogenous Mg^2+^ from the *E. coli* host strain (Fig. 4B). We propose a hydrolysis mechanism for Rexo2 activity, based on comparison of the structures of the two active sites in the Rexo2–RNA and Rexo2–DNA1 complexes (Fig. 4D; Supplemental Movie S1). Four DEDD residues—Asp47, Asp147, Asp199, and Glu49—coordinate two Mg^2+^ ions, which are further bound to the 3′-end scissile phosphate. His194 functions as the general base to activate a water molecule; we did not observe this water in all three complexes, likely because we used a low pH of 5.1 in the crystallization conditions, so His194 was in a protonated state not suitable for serving as a general base. Presumably, this His194-associated water (circled by a dashed line in Fig. 4D) attacks the scissile phosphate to generate a nucleoside monophosphate and a cleaved RNA with a 3′-OH end. Thus, Rexo2 hydrolyzes the phosphodiester linkage from the 3′-OH end by means of a two-Mg^2+^-ion-dependent catalytic mechanism.

## DISCUSSION

In this study, for the first time, we reveal the structural basis of how Rexo2 preferentially binds and degrades nanoRNAs of <5 nt to recycle their nucleoside monophosphates. Our results are applicable to Rexo2 homologs across species (including bacterial ORN) that share a high sequence identity, the same dimeric structure and similar enzymatic activities as Rexo2. Small nanoRNAs are bound by Rexo2 with a high affinity because Rexo2 primarily interacts with only the last two 3′-end nucleobases via hydrophobic and π−π stacking interactions. Consequently, Rexo2 preferentially degrades nanoRNAs without sequence specificity. Mitochondrial nucleases, such as PNPase, degrade large RNA molecules and produce small RNAs of <5 oligonucleotides. Rexo2 may further degrade these nanoRNAs into monoribonucleotides for RNA salvage (Nguyen et al. 2000). Rexo2 depletion can therefore result in decreased levels of mitochondrial RNA (Bruni et al. 2013), so Rexo2 plays an essential role in RNA metabolism in mitochondria.

**FIGURE 5.**
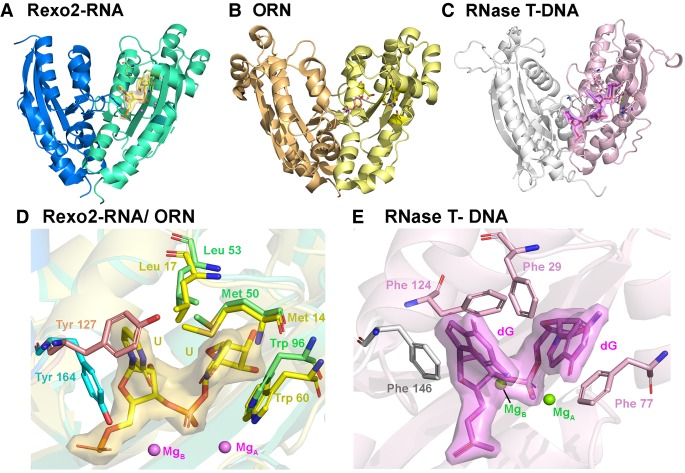
The crystal structure of Rexo2 strongly resembles that of ORN. (*A*) Crystal structure of Rexo2–RNA with the active-site residues and RNA shown in stick model. (*B*) Crystal structure of the *apo* form of ORN (PDBID :2IGI) from *E. coli*. (*C*) Crystal structure of RNase T bound with DNA (PDBID: 3NH1). (*D*) Superimposition of RNA-binding aromatic residues in the active site of Rexo2–RNA and ORN revealing that these residues are located at similar positions. (*E*) Stick representation of the two 3′-end DNA nucleobases (dGdG) that make π–π stacking interactions with the four aromatic Phe residues in the active site of RNase T.

Sequence alignment of various Rexo2 homologous proteins across species shows that these three hydrophobic and π−π interaction residues in Rexo2 (Leu53, Trp96, and Tyr164) are strictly conserved, demonstrating their crucial roles in substrate binding (Supplemental Fig. S2). We superimposed the Rexo2–RNA complex structure with those of two baterial homologs, ORN (PDBID: 2IGI) and RNase T-DNA complex (PDBID: 3NH1). Rexo2 has a high structural resemblance to ORN, with an average RMSD of 1.14 Å (for 319 Cα atoms), but a lower resemblance to RNase T (average RMSD of 7.64 Å for 319 Cα atoms). The three hydrophobic and aromatic residues in ORN (Leu17, Trp60, and Tyr127) are located at positions matching those of Rexo2, suggesting that they also participate in base stacking interactions with RNA substrates (Fig. 5D). In contrast, although RNase T interacts with the last two 3′-end nucleobases via π−π stacking interactions, it uses four phenylalanine residues, i.e. Phe29, Phe77, Phe124, and Phe146 (PDBID: 3NH1) (Fig. 5E; Hsiao et al. 2011, 2012, 2014). Therefore, based on the sequence alignment and structural comparisons, we conclude that Rexo2 is assembled into a homodimer, and it binds and degrades nanoRNAs in a manner that highly resembles that of bacterial ORN, providing strong evidence for a role for Rexo2 in ribonucleotide salvage in human mitochondria.

In this study, we also show that Rexo2 binds and degrades small DNA oligonucleotides in the presence of Mg^2+^. The crystal structures of Rexo2 in complex with DNA reveal a similar DNA oligonucleotide-binding mode to that for RNA. A number of lines of evidence suggest that Rexo2 is involved in DNA metabolism. For example, Rexo2 knockdown in HeLa cells results in decreased mitochondrial DNA levels (Bruni et al. 2013). Furthermore, Rexo2 is involved in cell resistance to UV-C radiation (Ito et al. 2007). Moreover, Rexo2 knockdown induces accumulation of cyclobutane pyrimidine dimers (Ito et al. 2007). Thus, it is likely that Rexo2 also participates in degrading the small DNA intermediates generated during DNA replication and/or repair pathways to recycle the resulting deoxynucleoside monophosphates.

Finally, it is intriguing that both Rexo2 and PNPase are primarily located in the mitochondrial intermembrane space and only a small amount of these proteins are located in the matrix (Wang et al. 2010; Bruni et al. 2013). Several endonucleases with high nucleic acid degradation activities, including EndoG (Lin et al. 2016a,b) and RNaseT2 (Liu et al. 2017), are also located in the mitochondrial intermembrane space, prompting the question if compartmentalization of these nucleases in this region is a means of restricting their nuclease activities or that mtRNAs are degraded in the intermembrane space. Here, we provide a solid structual basis for the nanoRNA scavaging mechanism used by Rexo2, but further studies are required to reveal how and where its activity is regulated and executed in mitochondria.

## MATERIALS AND METHODS

### Cloning, protein expression, and purification

The gene encoding human Rexo2 (residues 33–223) without the mitochondria localization sequence (residues 1–25), amino-terminal tail (residues 26–32) and carboxy-terminal tail (residues 224–237) was amplified by PCR using PfuUltra II (Agilent Techonologies), and the PCR products were subcloned into the NdeI/SalI sites of the pET28a vector (Invitrogen) with an amino-terminal 6xHistidine tag added to the construct. Plasmids encoding the Rexo2–D199A and Rexo2–H194A mutants were generated using QuickChange site-directed mutagenesis kits (Stratagene) from the wild-type construct. All plasmids were transformed into *E. coli* host BL21-CodonPlus (DE3) RIPL cells and incubated at 37°C overnight in LB media supplemented with 50 μg/mL Kanamycin. The cells were then transferred into 1 L of LB medium and grown until the OD_600_ reached 0.6. Rexo2 protein expression was induced at 18°C for 18 h by 0.8 mM IPTG. Cells were harvested and disrupted by Microfluidizer (Microfluidics M-110P) in buffer A containing 50 mM Tris–HCl pH 8.0, 1 M NaCl, 5 mM imidazole, 10 mM β-mercaptoethanol. Supernatants were applied to HisTrap HP columns (GE HealthCare) and the bound proteins were eluted using an imidazole gradient between buffer A and buffer B (20 mM Tris–HCl pH 8.0, 1 M NaCl, 500 mM imidazole). The eluted protein fractions were collected and Thrombin (1 unit/mL) was added, and the mixture was dialyzed into a buffer (50 mM Tris–HCl pH 8.0, 150 mM NaCl, 10 mM β-mercaptoethanol) overnight to remove the amino-terminal His-tag. Recombinant proteins were loaded again into HisTrap HP columns (GE HealthCare), and the flow-through fractions were collected to recover His-tag-free Rexo2. The recombinant Rexo2 protein samples were concentrated and purified by Superdex 75 10/300 GL columns (GE HealthCare) in a running buffer of 20 mM Tris–HCl pH 7.5, 150 mM NaCl, and 2 mM DTT. The purified protein samples were stored at −20°C or concentrated to 20 mg/mL for crystallization.

### RNA and DNA degradation assays

Wild-type Rexo2, Rexo2–D199A, or Rexo2–H194A (1 μM for RNA degradation assays, 2 µM for DNA degradation assays) was incubated with 2.5 nM 5′-end-^32^P-labeled ssRNA (5′-^32^P-A_4_-3′ or 5′-^32^P- A_12_-3′) at 37°C for 0–60 min in a reaction buffer containing 50 mM Tris–HCl (pH 7.5), 50 mM NaCl, 1 mM DTT, and 5 mM MgCl_2_ in a final reaction volume of 10 µL. ORN from *E. coli* was used as a control, with final concentrations of ORN in the assays being 50 nM for RNA degradation and 2 µM for DNA degradation. The reactions were stopped by adding 2× urea loading dye (Thermo Fisher Scientific) at different time-points (0, 5, 15, 30, and 60 min), and the samples were loaded on 7.5 M urea/20% (w/v) polyacrylamide denaturing electrophoresis gels. All control reactions were stopped at the 60 min time-point. RNA degradation patterns were exposed on a FujiFilm Image plate and detected by Typhoon FLA 9000 (GE HealthCare). The same procedures were also applied for DNA degradation assays, with the DNA substrate being 5′-end-^32^P-labeled ssDNA (5′-^32^P-T_4_ -3′ or 5′-^32^P- T_12_-3′).

### RNA and DNA binding assays

The RNA binding affinity of Rexo2 was measured by assessing changes in fluorescence polarization signals using a Paradigm plate reader (Molecular Devices). The single-stranded RNA substrates—2-nt RNA (5′-Cy3-A_2_-3′), 5-nt RNA (5′-Cy3-A_5_-3′) and 11-nt RNA (5′-Cy3-AGCGCAGUACC-3′)—were labeled at the 5′-hydroxyl end with Cyanine-3 (Dharmacon). These RNA substrates (10 nM) were titrated with the indicated concentrations of the Rexo2–H194A mutant in binding buffer (50 mM Tris–HCl pH 7.5, 50 mM NaCl, and 50 mM EDTA). Rexo2 and RNA were incubated for 15 min at room temperature (25°C), and the fluorescence polarization signals were excited at 535 nm and read at 595 nm. The RNA-binding affinities of Rexo2 were calculated by fitting the binding curve to a one-site-binding Hill slope using the software GraphPad PRISM 7. For DNA-binding assays, 3-nt DNA (5′-Cy3-T_3_-3′), 5-nt DNA (5′-Cy3-T_5_-3′), and 12-nt DNA (5′-Cy3-T_12_-3′) substrates were labeled at the 5′-hydroxyl end with Cyanine-3 (MD Bio), and the same fluorescence polarization method as used for the RNA-binding assays was applied.

### Crystallization and structural determination

All three Rexo2-oligonucleotide complexes were crystallized by the hanging-drop vapor diffusion method at room temperature in similar conditions by mixing 1 µL of protein-oligonucleotide mixture (protein to oligonucleotide ratio of 1:1.2) with 1 µL of reservoir solution (Hampton PEG/Ion Screen, number 48: 0.2 M ammonium citrate dibasic, 20% w/v polyethylene glycol 3350 pH 5.1). X-ray diffraction data were collected at BL-15A1 beamline at the National Synchrotron Radiation Research Center (NSRRC), Hsinchu, Taiwan, and the collected data were processed and scaled by HKL2000. Rexo2 structures were determined using the AutoMR function in the program Phenix, using the *E. coli* ORN structure (PDBID: 2IGI) as the search model. The oligonucleotide structural model was built using Coot and the Rexo2-oligonucleotide structures were refined in Phenix.

## DATA DEPOSITION

Structural coordinates and diffraction structure factors of Rexo2–RNA, Rexo2–DNA1, and Rexo2–DNA2 have been deposited in the RCSB Protein Data Bank with the PDB ID codes 6J7Z, 6J7Y, and 6J80, respectively.

## SUPPLEMENTAL MATERIAL

Supplemental material is available for this article.

## Supplementary Material

Supplemental Material
