# Supplementary material for: Structural insights into nanoRNA degradation by human Rexo2
Source: RNA. 2019 Jun;25(6):737–46. doi: 10.1261/rna.070557.119 (PMC6521605; doi:10.1261/rna.070557.119)
Supplement: Supplemental Material [file supp_25_6_737__index.html]

Structural insights into nanoRNA degradation by human Rexo2 — Supplemental Material 

# Structural insights into nanoRNA degradation by human Rexo2

## Supplemental Material

- Supplemental\_Figures\_Legends.pdf
- Supplemental\_Movie\_S1.mp4
