# Supplementary material for: Structural insights into nanoRNA degradation by human Rexo2
Source: RNA. 2019 Jun;25(6):737–46. doi: 10.1261/rna.070557.119 (PMC6521605; doi:10.1261/rna.070557.119)
Supplement: Supplemental Material [file supp_070557.119_Supplemental_Figures_Legends.pdf]

Supplementary Information

Structural insights into nanoRNA degradation by human Rexo2

Lee-Ya Chu[1,2,3&], Sashank Agrawal[1,4,5&], Yi-Ping Chen[1], Wei-Zen Yang[1] and Hanna S. Yuan[1*]

This document includes:

Supplementary Figures S1 and S4

Caption for Supplementary Movie S1

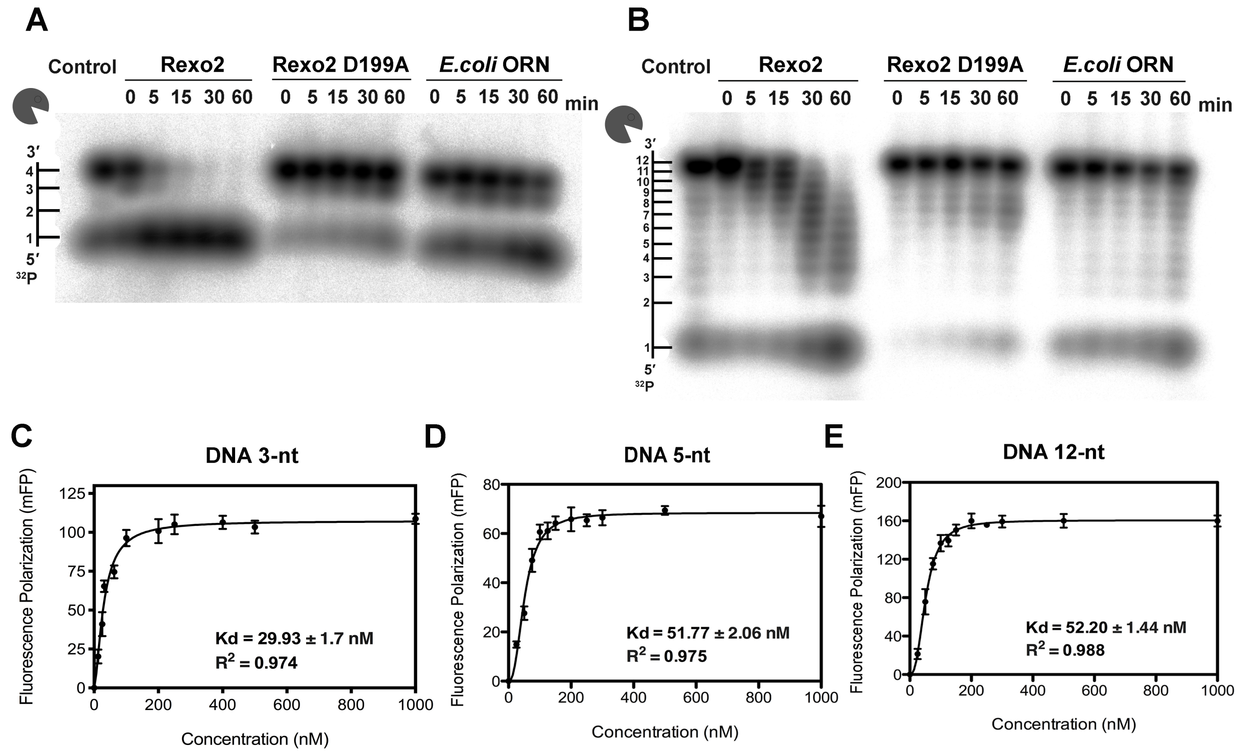

Supplementary Figure S1. Rexo2 binds and degrades single-stranded DNA. (A-B) Rexo2 (2 μM) degraded a 4-nucleotide DNA (5'-P^{32}-T_4-3', 2.5 nM) and a 12-nucleotide DNA (5'-P^{32}-T_{12}-3', 2.5 nM) in the presence of 5 mM $MgCl_2$, but Rexo2 D199A mutant (2 μM) lost the DNase activity. *E. co*li ORN (2 μM) had only residual activity in degrading DNA. (C-E) DNA-binding affinities between Rexo2 H194A and 3-nt DNA (5′-Cy3-T_3-3′), 5-nt DNA (5′-Cy3-T_5-3′) and 12-nt DNA (5′-Cy3-T_{12}-3′) substrates were measured by fluorescence polarization (in mFP units) and plotted against protein concentrations.

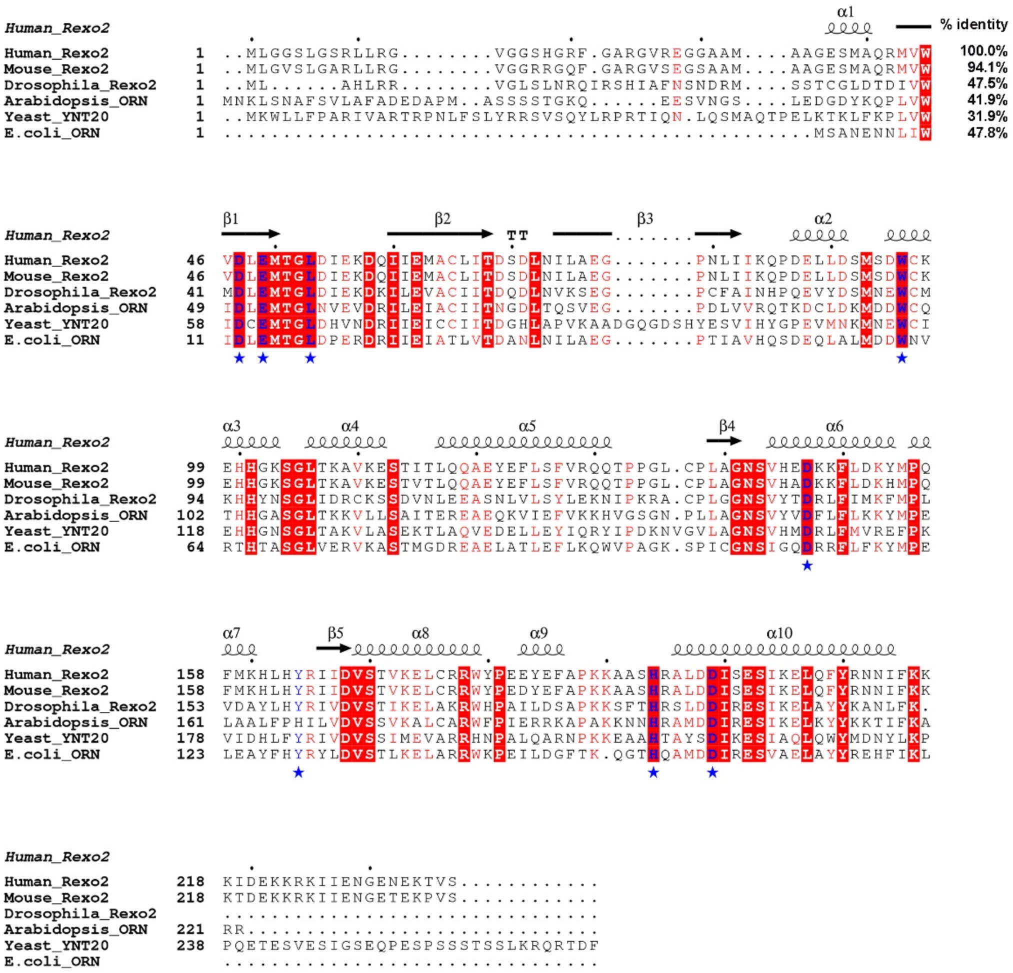

Supplementary Figure S2. Sequence alignment of Rexo2 with ORN homologs reveals that the catalytic residues Asp47, Asp147, Asp199 and Glu49, and nucleobase-stacking residues, Leu53, Trp164 and Tyr96, are all strictly conserved. The secondary structures of α-helices (coils) and β-stands (arrows) revealed by the crystal structure of human Rexo2 are shown above the sequence alignment. Sequence identities between human Rexo2 and ORN homologs are listed at the top right corner.

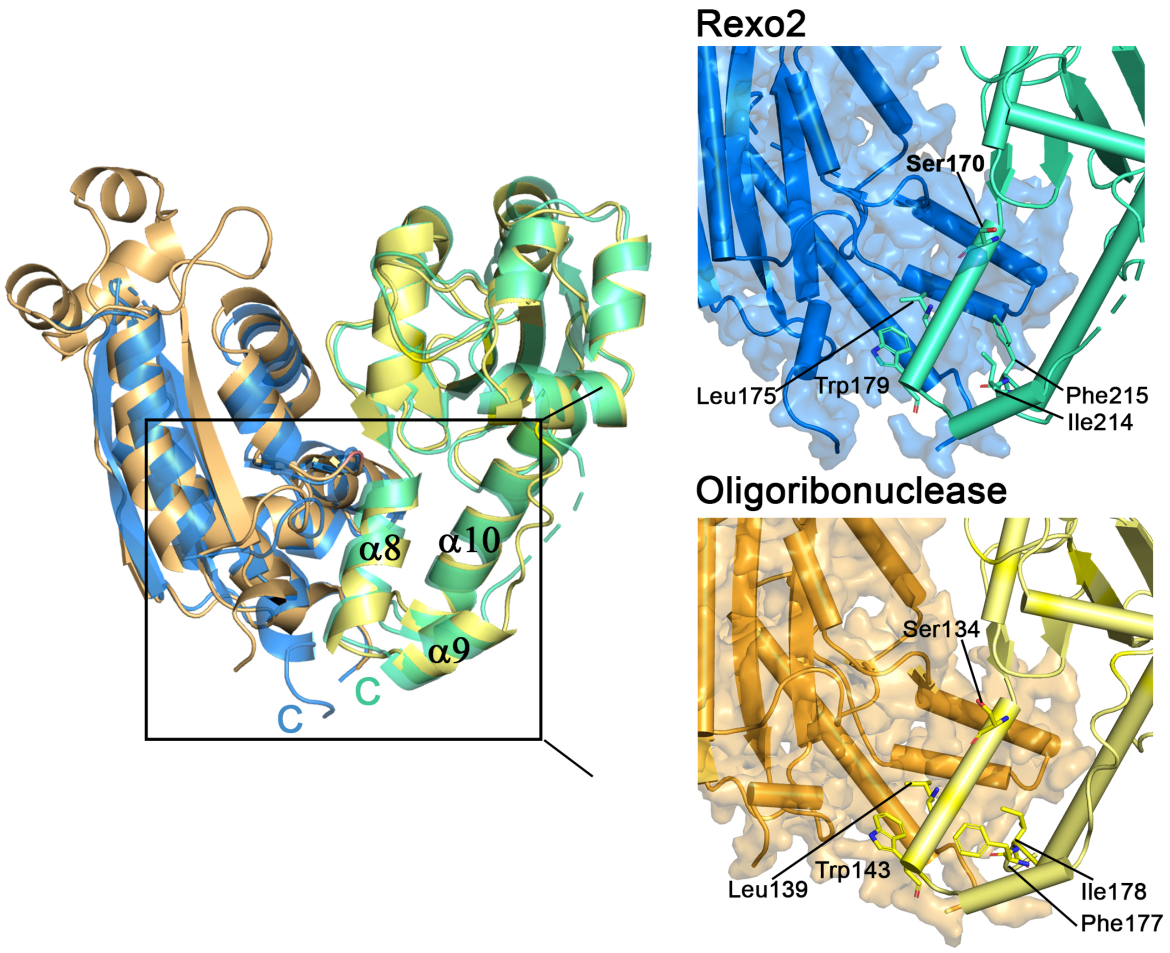

Supplementary Figure S3. Dimerization interfaces in Rexo2 and Oligoribonuclease (Orn) are highly conserved. Structure alignment between Rexo2 (this study, PDB entry: 6J7Z) and *E. coli* Orn (PDB entry: 2IGI) reveals that both proteins are homodimers with similar dimerization interfaces formed by three α-helices, α8, α9 and α10 (left panel). The dimeric interfaces in Rexo2 contain hydrophobic residues, including Ser170, Leu175, Trp179, Ile214 and Phe215 (top right panel), and these residues are conserved in the interfaces of ORN dimer (Ser134, Leu139, Trp143, Phe177 and Ile178, bottom right panel).

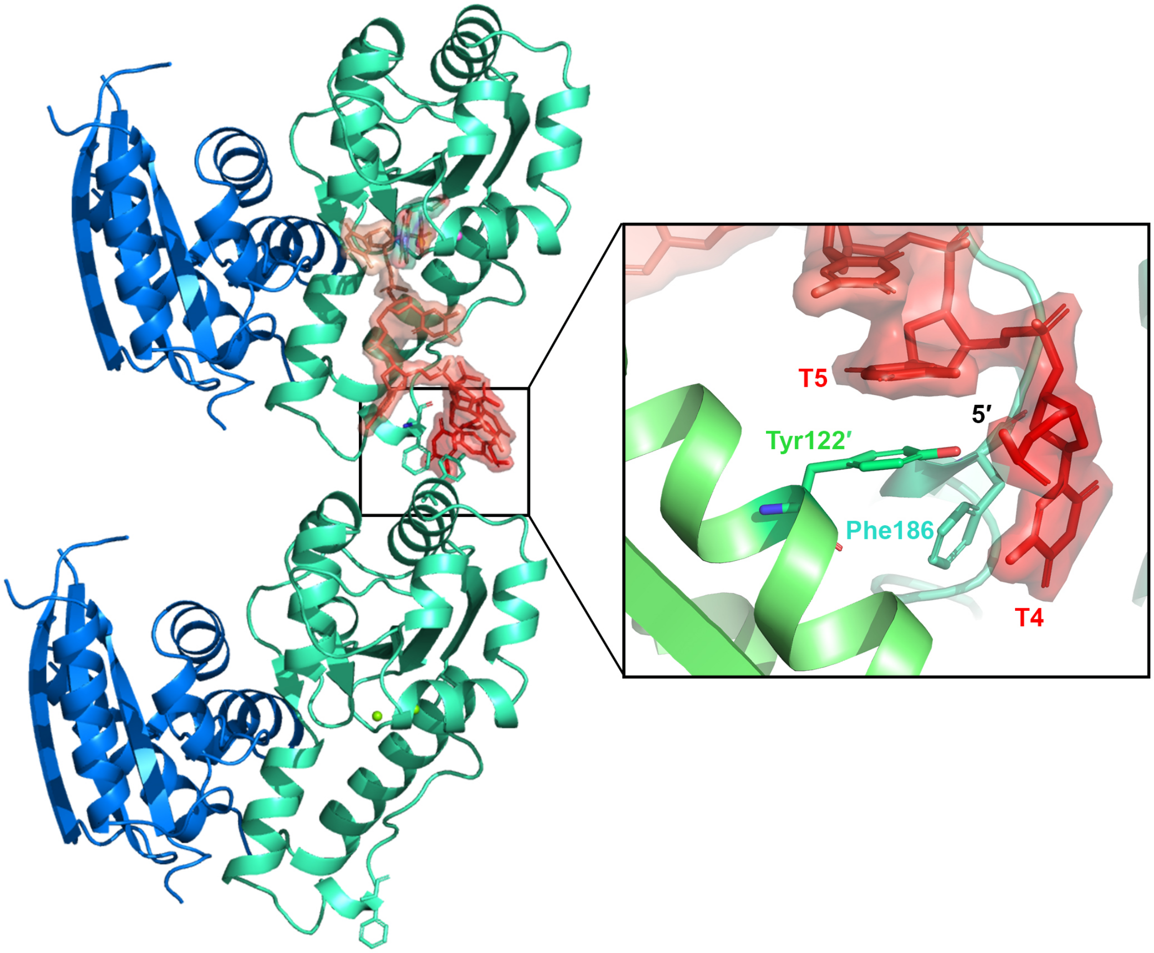

Supplementary Fig. S4. The long DNA in the Rexo2-DNA2 complex also makes π-π stacking interactions with the neighboring Rexo2 protein. Seven nucleotides (T4-T10) were observed in Rexo2-DNA2 complex (PDB entry: 6J80) with a schematic diagram for Rexo-DNA interactions listed in Fig. 3D. The π-π stacking interactions were observed between T4 nucleobase and Phe186 (see the enlarged right panel). Due to crystal packing, the π-π stacking interactions were also formed between T5 nucleobase and Tyr122 of the neighboring Rexo2 molecule (labeled as Tyr122' in the right panel).

Supplementary Movie S1. **How Rexo2 binds and degrades nanoRNA.**

Crystal structures of Rexo2 reveal that Rexo2 forms a homodimer and interacts mainly with the last two 3′-end nucleobases of substrates by π–π stacking interactions via Trp96 and Tyr164 which further stack with His100 and His163, respectively. Four DEDD residues in the active site—Asp47, Asp147, Asp199 and Glu49—coordinate two Mg^{2+} ions, which are further bound to the 3′-end scissile phosphate. His194 functions as the general base to activate a water molecule to attack the scissile phosphate and generate the cleavage products, a nucleoside monophosphate and a cleaved RNA with a 3′-OH end.
